# Protocol for using Ciclops to build models trained on cross-platform transcriptome data for clinical outcome prediction

**DOI:** 10.1016/j.xpro.2022.101583

**Published:** 2022-07-20

**Authors:** Elysia Chou, Hanrui Zhang, Yuanfang Guan

**Affiliations:** 1 Department of Computational Medicine and Bioinformatics, Michigan Medicine, University of Michigan, Ann Arbor, MI 48109, USA; 2 Department of Internal Medicine, Michigan Medicine, University of Michigan, Ann Arbor, MI 48109, USA

**Keywords:** Bioinformatics, Clinical Protocol, Genomics, Systems biology

## Abstract

Designing robust, generalizable models based on cross-platform data to predict clinical outcomes remains challenging. Building explainable models is important because models may perform differently depending on the conditions of the samples. Here, we describe the use of Ciclops (cross-platform training in clinical outcome predictions), freely available software that can build explainable models to deliver across cross-platform datasets for predicting clinical outcomes. This protocol also utilizes SHAP, a post-training analysis allowing for assessing potential biomarkers of the clinical outcome under study.

For complete details on the use and execution of this protocol, please refer to Zhang et al. (2022).

## Highlights

• Build robust clinical outcome prediction models using cross-platform transcriptome data
• Applicable to datasets from different studies measuring different clinical outcomes
• Perform key preprocessing steps of imputation and cross-platform quantile normalization
• Analyze feature importance in LightGBM, XGBoost, and Random Forest models with SHAP

## Abstract (editor highlights)

Publisher’s note: Undertaking any experimental protocol requires adherence to local institutional guidelines for laboratory safety and ethics.

## Graphical abstract

**Figure:**
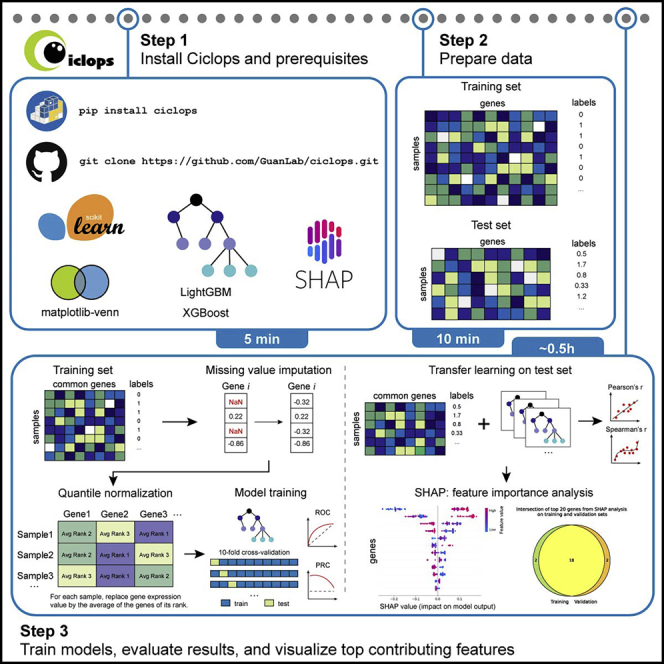

## Before you begin

Consistency and reproducibility are key to the generalizability of using transcriptomic data to predict clinical outcomes. Datasets that differ with regards to experimental platforms, measurement targets, geographic sampling sites, and timing of sample collection present practical challenges to building transferable prediction models. Being able to leverage cross-platform datasets when building clinical prediction models would not only allow for widespread applicability of the model, but also increase our confidence in the robustness of the biomarkers that result from feature importance analysis.

Studies analyzing the consistency of various microarray platforms have been conducted for as long as microarrays have been in use (Guo et al., 2006; Fan et al., 2010), providing us with preprocessing methods to consider before attempting to build cross-platform prediction models. Proper preprocessing, cross-platform normalization, and appropriate selection of machine learning methods can lead to novel scientific discoveries, even when integrating microarray and RNAseq data (Fauteux et al., 2021). With the wealth of transcriptome data being produced every year, developing more approaches to integrate various relevant datasets across platforms and across data collection methods carries the potential to uncover new scientific insights.

Here, we present our protocol for using Ciclops (Cross-platform training In Clinical Outcome PredictionS) to build predictive models trained on transcriptome data. These models are then evaluated on external datasets the user provides. Our package is versatile, easy to install, and straightforward to use with a one-line command in the terminal. Our package’s pipeline, which was central to winning the 2019 Malaria DREAM Challenge (Sage Bionetworks, 2018), performs imputation and quantile normalization on the datasets before model construction. Additionally, Ciclops allows users to investigate the top features contributing to model performance using SHAP analysis (Lundberg and Lee 2017). With SHAP, researchers can build explainable models and assess the significance of certain biomarkers of the clinical outcome under study.

### Software prerequisites and data requirements

Ciclops can be run on Linux and Mac operating systems with Python 3. Before launching Ciclops, ensure your versions of Python (>=3.8), LightGBM (>=3.3.2) and XGBoost (>=1.6.0) meet the minimum version requirements (see key resources table). It is also recommended that users have Conda installed.

Users should also prepare the transcriptomic datasets they wish to analyze using Ciclops, as well as the labels for the clinical outcomes they wish to study. While Ciclops can be applied to datasets from different studies measuring different clinical outcomes, here we illustrate how to use Ciclops using two datasets from GEO, which can be downloaded by using the script in https://github.com/GuanLab/ciclops/tree/main/external_data. Both example datasets were used in our previous analysis (Zhang et al., 2022) and contain binary labels for fast or slow clearance rate of the parasite under study. While it is not necessary to download this data, it may be of interest to test whether Ciclops works as expected in your local environment. While this example only uses binary classification, Ciclops can also be used for multiclass classification or regression analysis.

### Create a virtual environment for your project (recommended)

**Timing: 5 min**

While this step is optional, it is recommended that users create a virtual environment to install and run Ciclops to ensure following the protocol goes smoothly.

1. Create a new environment and specify a version of Python that Ciclops is compatible with:

conda create--name [ENV_NAME] python=3.8

where you can enter the name of your environment in the place of the square brackets. You can also install Ciclops’ dependencies in this step, such as *numpy* and *scikitlearn* (see key resources table).

2. When you are ready to use this protocol, activate the environment:

conda activate [ENV_NAME]

and use the command:

conda deactivate

when you wish to deactivate this environment.

## Key resources table

**Table:** 

REAGENT or RESOURCE	SOURCE	IDENTIFIER
**Software and algorithms**		

Python (>=3.8)	Python Software Foundation	https://www.python.org/downloads/release/python-3812/
numpy (>=1.21.5)	Harris et al. (2020)	https://pypi.org/project/numpy/
pandas (>=1.4.1)	McKinney (2010)	https://pandas.pydata.org/
scikit-learn (>=1.0.2)	Pedregosa et al. (2011)	https://scikit-learn.org/stable/
scipy (>=1.8.0)	Virtanen et al. (2020)	https://scipy.org/
matplotlib (>=3.5.1)	Hunter (2007)	https://matplotlib.org/
matplotlib-venn (>=0.11.7)	Konstantin Tretyakov	https://pypi.org/project/matplotlib-venn/
lightgbm (>=3.3.2)	Microsoft Corporation	https://lightgbm.readthedocs.io/en/latest/
shap (>=0.40.0)	Lundberg and Lee (2017)	https://shap.readthedocs.io/en/latest/index.html
xgboost (>=1.6.0)	Chen and Guestrin (2016)	https://xgboost.readthedocs.io/en/stable/
GEOparse (>= 2.0.3)	Rafal Gumienny	https://geoparse.readthedocs.io/en/latest/index.html
tqdm (>=4.63.0)	da Costa-Luis (2022)	https://github.com/tqdm/tqdm
Ciclops	This paper	https://pypi.org/project/ciclops/ or https://github.com/GuanLab/ciclops (https://doi.org/10.5281/zenodo.6686200)

## Materials and equipment

A minimum of 16 GB local memory is recommended (see Table 1 for computational resources used in this study). However, for larger datasets, you may want to run Ciclops on a computing cluster with multiple cores and larger RAM to shorten the run time. Since Ciclops was developed on an Ubuntu Linux system with Python 3 (>=3.8), it is recommended to run Ciclops on a Linux machine (Linux, Mac OS, or Windows Subsystem for Linux).

**Table 1:** Computational resources used in this study

Operating system	Version
Ubuntu	20.04.3 LTS (Focal Fossa)

**CPU Information**	**Value**

RAM	16 GB
Cores	6
Processor speed	1.1 GHz

## Step-by-step method details

### Download Ciclops and install prerequisites

**Timing: 5 min**

**Figure 1:**
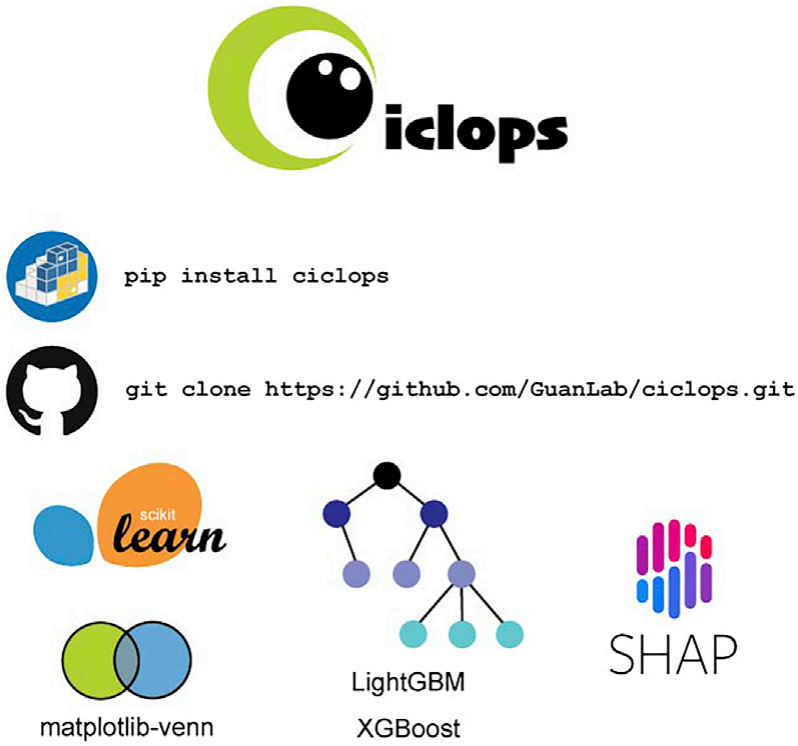
Model installation using pip or git and the main dependencies

Obtain the latest version of Ciclops, which can be found on PyPI or our GitHub (Figure 1).

1. Install Ciclops via pip:

pip install ciclops

or use the following command to clone the protocol directory from our GitHub repository to your local directory:

git clonehttps://github.com/GuanLab/ciclops.git

**CRITICAL:** Refer to the key resources table to ensure your local environment is compatible with all the package’s dependencies, in case installation errors occur (troubleshooting 1).

***Optional:*** Once installed, you can run the following command to see if Ciclops was installed properly:

ciclops--help

If any errors or warnings occur due to clashing package versions, see troubleshooting 2.

Additionally, if you are cloning the GitHub repository, you can download the example data from GEO using the Python script in the external_data/ directory:

python3 getGEO.py

This script obtains two transcriptomic datasets: 1) *in vitro* data of P. falciparum (GEO: GSE151189) (Mok et al., 2021), used as the training set in our example; and 2) *ex vivo* data of P. falciparum from a different study (GEO: GSE59098) (Mok et al., 2015), used as the test set in our example. In this protocol, these data are used only for illustrative purposes.

### Prepare data

**Timing: 10 min**

In this step, format the data so that it will be suitable for use with Ciclops (Table 2). The data should be in csv format.

2. Select datasets and preprocess the data.
  a. Select at least two transcriptomic datasets that you wish to build a predictive model with: one training set and at least one test/validation set.
  b. Ensure the labels are somewhat consistent between the training and test set based on the biological context of the problem you are trying to solve.

***Note:*** As an example, in our original study, the training set’s labels were fast or slow clearance rate of the parasite after introduction of the drug in question and the test set’s labels were the drug’s IC50. Since lower IC50 should correspond to faster clearance rate, ‘fast’ was labeled as 0, and ‘slow’ was labeled as 1.

3. Format both datasets such that the features and labels are arranged correctly (Table 2).
  a. Ensure the columns are gene expression levels for each gene and that the rows are samples.
  b. Put the sample names or numbers in the first column.
    i. If the datasets being used do not contain sample numbers, put a column of numbers in the first column as a placeholder.
  c. Put the labels in the last column.

**CRITICAL:** 1.) All datasets used with Ciclops should follow the format above, as Ciclops assumes the first and last column are sample numbers and labels respectively. 2.) While these datasets can be collected from different platforms and/or use different clinical outcome metrics (e.g., the training set and test set are transcriptome profiles measured using different platforms; or the labels are different metrics for measuring a similar clinical outcome), they must share a set of common genes in order to build a model (troubleshooting 3).

***Optional:*** 1.) If the datasets you have selected still contain raw data, preprocess both training and test data, following standard protocol according to their respective platforms. For transcriptomic datasets, this often involves feature extraction, QC, and some form of normalization, among other preprocessing steps. For example, while we use the processed data from GEO as our example files, we could have also elected to use the raw data the authors provided instead. In that case, we would have preprocessed the datasets individually as described in the methods of our original study (Zhang et al., 2022). Subsequently, Ciclops will perform imputation and cross-platform quantile normalization to normalize the training and test data with respect to each other. 2.) In the case of missing values in the datasets, it is recommended that researchers enter ‘NaN’ in those positions rather than leaving them blank. 3.) At this stage, imputing missing values is not necessary, as Ciclops performs gene-wise imputation before training the models, i.e., a sample’s missing value is replaced with the relevant gene’s average expression level. Users can perform different imputation methods in this step if they so wish.

**Table 2:** Example data format for both training and test sets

Sample	Gene 1	Gene 2	…	Gene p	Label
1	0.15	0.39	…	-2.05	0
2	0.01	-0.47	…	-1.14	1
…	…	…	…	…	…
N	0.53	-1.12	…	0.29	0

Note that the label can be categorical or continuous.

### Train models, evaluate results, and visualize top contributing features

**Timing: ∼ 0.5 h (depending on data size and model)**

**Figure 2:**
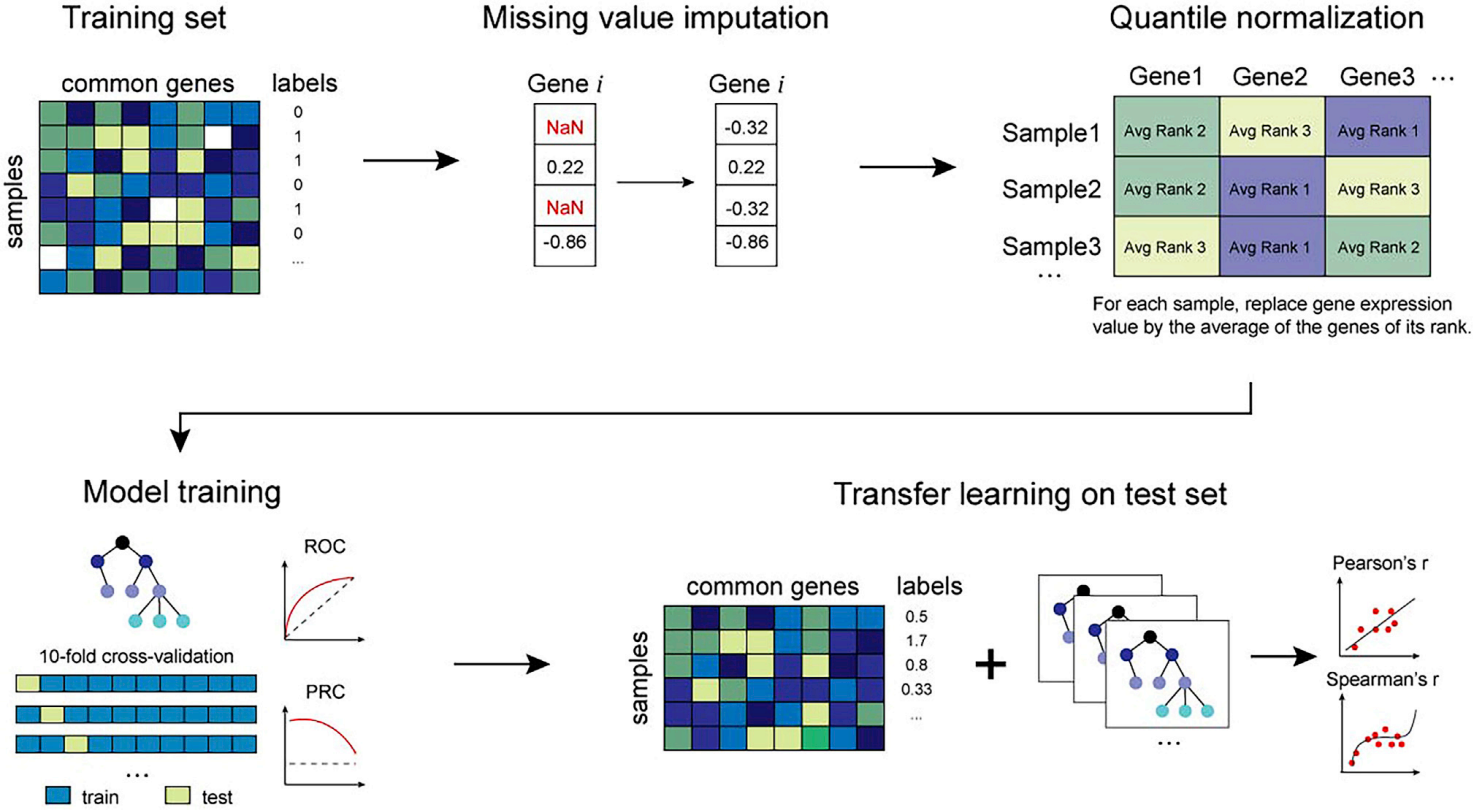
Workflow Using a one-line command, Ciclops first preprocesses both training and testing datasets by performing gene-wise imputation and quantile normalization. The program then trains the specified machine learning model using ten-fold cross-validation. Finally, it evaluates these models on the provided test set. This workflow does not include SHAP analysis.

With a one-line command, Ciclops allows the user to preprocess the data (imputation and quantile normalization), perform ten-fold cross-validation on the training set, and perform the transfer learning on the test set (Figure 2). Additionally, if specified, Ciclops will carry out SHAP analysis with a visualization report of top contributing features for both training and test sets.

4. Run Ciclops using the following command:
  ciclops--train_path [TRAIN_PATH]--valid_path [VALID_PATH] -m [MODEL_TYPE]--no_quantile--shap -n [TOP_GENES]
  The arguments are defined as follows:
  a. --train_path [TRAIN_PATH]: the path to the csv file containing the training data prepared in the previous steps. For example, if using the example data, enter the path to the file in_vitro_GSE151189.csv.
  b. --valid_path [VALID_PATH]: the path to the csv file containing the transfer validation or test data prepared in the previous steps. For example, if using the example data, enter the path to the file ex_vivo_GSE59098.csv.
  c. -m [MODEL_TYPE]: the machine learning model to use. The default is LightGBM; however, the user can specify that one of the following models be used instead, using the specified argument:
    i. lgb: LightGBM.
    ii. xgb: XGBoost.
    iii. rf: Random Forest.
    iv. gpr: Gaussian Process Regression.
    v. lr: Linear Regression.
  d. --no_quantile: if this argument is used, Ciclops will not perform quantile normalization on the training and test data. The user should exclude this argument if they wish to perform quantile normalization on the data.
  e. --shap: if this argument is specified, Ciclops will perform SHAP analysis on the training and test data.
  f. -n [TOP_GENES]: if using the --shap argument, users can set the number of top-contributing genes to compare between the training and test set with the -n flag. The default is 20.
    ***Note:*** 1.) When deliberating what model type to use, LightGBM would generally be a good place to start since it performs well in our experience and is very efficient for training large datasets such as transcriptomic data that Ciclops is intended for (Ke et al., 2017). While XGBoost is not as fast in that regard (Ke et al., 2017), comparing your LightGBM model to models trained by XGBoost and Random Forest can give you insight into the performance of tree-based learners versus kernel-based algorithms such as GPR and linear regression, which have more straightforward interpretability. 2.) If testing Ciclops with the example data, which is relatively small, running the command in step 4 should take no more than five minutes. Since we anticipate Ciclops being used for much larger datasets, as was done in our original study, the timing estimate given above is based on our original study. As mentioned in the materials and equipment section, running Ciclops on a computing cluster with multiple cores and larger RAM will shorten the runtime. 3.) Unless both datasets have been jointly normalized during your preprocessing step, it is recommended to exclude the --no_quantile argument, as quantile normalization played a key role in building a successful prediction model in our original publication. 4.) In Ciclops, SHAP can only be used with tree-based models (i.e., LightGBM, Random Forest, XGBoost) as that is mainly what SHAP is designed for. It is infeasible to perform SHAP analysis on regression models with the thousands of features that transcriptome data usually comes with. 5.) At the time of writing, the *shap* package utilizes the *IPython.core.display* module for creating interactive plots. Since storing SHAP values and generating SHAP summary plots was sufficient in our original study, Ciclops only makes use of the non-interactive plot functions of the *shap* package. Therefore, if applicable, users can ignore the warning, "IPython could not be loaded!" and do not need to have IPython installed.
    **CRITICAL:** 1.) In order for Ciclops to run, ensure the paths to both training and test data files are correct. 2.) Unless you intend on using a tree-based model (e.g., LightGBM, Random Forest, XGBoost), exclude or minimize the number of features that are categorical, as most models implemented in Ciclops were designed to operate on ordinal, numeric values. If you wish to include categorical features, encode them as ordinal, numeric values before training. 3.) While the parameters of the five models implemented in Ciclops worked for our original study and allowed us to win the 2019 Dream Malaria challenge (Table 3), we encourage users to tune the hyperparameters in order to obtain the best model fit for their datasets (see troubleshooting 4). 4.) While both example datasets use binary labels, Ciclops can also build models where the labels of one or both datasets are continuous values. Use evaluation metrics that are suitable to assess the outcomes you are trying to predict with your model (see quantification and statistical analysis).
    **Table 3:** Parameters used in the different machine learning models

Parameter	Value	Description
**LightGBM**		

boosting_type	‘gbdt’	The gradient boosting method to use.
objective	‘regression’	The learning task (regression, binary classification, etc.).
num_leaves	5	The maximum number of leaves in a tree.
learning_rate	0.05	The learning rate for the gradient boosting model.
Verbose	0	The level of verbosity, mainly for debugging.
n_estimators	800	The number of boosted trees to build in order to improve the fit.
reg_alpha	2.0	The L1 regularization term for combatting overfitting.

**XGBoost**		

N/A	Default	N/A

**Random Forest**		

max_depth	2	The maximum tree depth.
n_estimators	100	The number of trees to use in the model.

**Gaussian Process Regression (GPR)**		

Kernel	DotProduct() + WhiteKernel()	Defines a Gaussian process by describing the covariance of the Gaussian process random variables. To explore the kernels that can be used with GPR, please refer to the *scikit-learn* documentation.

**Linear Regression**		

N/A	Default	N/A

## Expected outcomes

After running Ciclops using the command in step 4, the results can be found in the following newly-created subdirectories.

The first directory listed will be the training directory. This directory contains the train and test splits for each fold of the ten-fold cross-validation performed on the training dataset. As for the predicted labels for each sample in the test dataset, they are in the directory called validation. Next, the models generated from the ten-fold cross-validation are saved in the params directory. These saved models, named fold_∗_model.sav, can be reloaded using *pickle*, a standard module in Python. The fourth directory, called performance, contains the models’ evaluation results from each cross-validation fold for both training and test sets, as well as the results with a 95% confidence interval obtained by bootstrapping.

**Figure 3:**
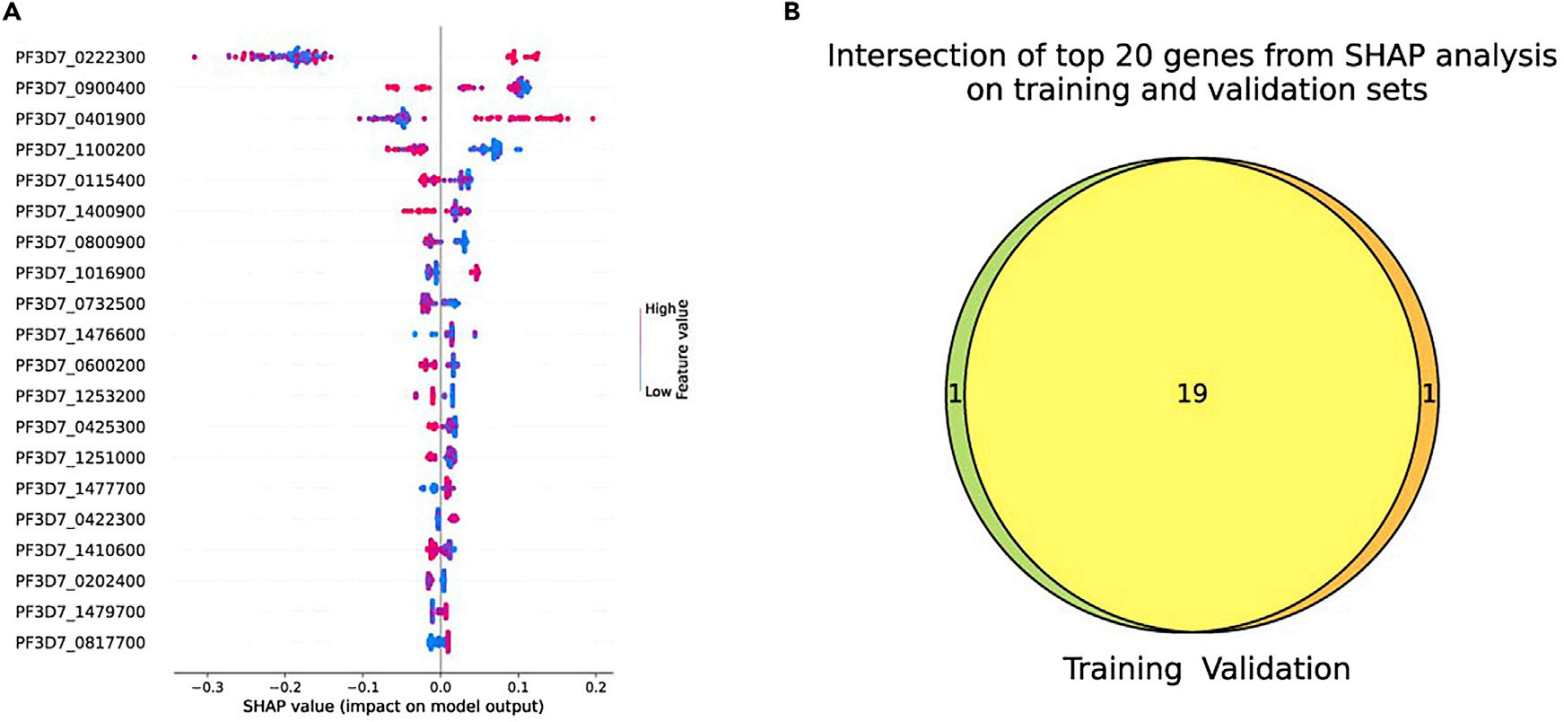
Results from SHAP analysis after running Ciclops on the example data (A) Beeswarm plot of the top 20 genes contributing to the model, with their respective SHAP values. (B) Venn diagram of the top 20 genes resulting from the SHAP analysis on both the training set and test/validation set, showing how many overlap.

Finally, if the --shap argument is specified, the results from the SHAP analysis will be stored in the SHAP directory. In this directory, the training and validation subdirectories contain figures with the top contributing genes and their SHAP values (SHAP_importance_∗.pdf) (Figure 3A) as well as csv files containing all genes and their SHAP values, for each cross-validation fold. The figure named intersection_venn_top_∗_genes.pdf displays a Venn diagram of the top contributing genes in the training and test/validation datasets, and shows how many of these genes overlap (Figure 3B). The last file you will find will be named intersection_list_top_∗_genes.txt. It lists the top contributing genes that overlap between the SHAP analysis done on the training set and the SHAP analysis done on the test/validation set.

When reviewing these results, verify that the performance results make sense; for example, the correlation coefficients are not NaN or the AUROC is higher than 0.5 (troubleshooting 5).

## Quantification and statistical analysis

Ciclops reports four metrics to evaluate model performance: AUROC, AUPRC, Pearson’s r, and Spearman’s ρ. They each have their own applications. For binary classification problems, you can look at the following:

• **AUROC** (Area Under the Receiver Operating Characteristic curve): The AUROC is a measure of the model’s ability to differentiate between classes, generally ranging in value from 0.5 (random classifier) to 1.0 (perfect classifier). In statistics terms, AUROC is the area under the ROC curve, which plots the true positive rate (TPR) against the false positive rate (FPR) across different decision thresholds:

$$TPR=\frac{TP}{TP\;+\;FN}\text{;}$$

$$FPR=\frac{FP}{FP+TN}$$

TP: True Positive; TN: True Negative; FP: False Positive; FN: False Negative.

In Ciclops, the AUROC is computed using the *sklearn.metrics* module in Python.

• **AUPRC** (Area Under the Precision-Recall Curve): The AUPRC is generally used when dealing with highly imbalanced datasets. The baseline AUPRC is given by the proportion of true positive samples in the dataset and an AUPRC closer to 1.0 (correlating with high precision and high recall) is preferred. The AUPRC measures the area under the PR curve, which plots the precision against the recall (equivalent to TPR) across different decision thresholds:

$$\mathrm{Precision}\;=\frac{TP}{TP\;+\;FP}$$

$$\mathrm{Recall}\;=\frac{TP}{TP\;+\;FN}$$

In Ciclops, the AUPRC is computed using the *sklearn.metrics* module in Python.

For regression problems, you can use the following of the reported metrics:

• **Pearson’s r** (Pearson correlation coefficient): the Pearson correlation coefficient measures the linear correlation between the predicted values and the actual values. The correlation coefficient is the ratio between the covariance and the product of the respective standard deviations:

$$r=\frac{\sum \left(x_{i}-\bar{x}\right)\left(y_{i}-\bar{y}\right)}{\sqrt{\sum {\left(x_{i}-\bar{x}\right)}^{2}{\left(y_{i}-\bar{y}\right)}^{2}}};$$

*x*_*i*_: predicted value of sample i;

$\bar{x}$: mean of the prediction values;

*y*_*i*_: actual value of sample i;

$\bar{y}$: mean of the actual values.

The Pearson correlation coefficient varies in range from -1 to 1. Both extremes indicate a strong linear correlation, while 0 indicates no correlation. In Ciclops, Pearson’s r is computed using the *scipy.stats* module in Python.

• **Spearman’s ρ** (Spearman rank-order correlation coefficient): The Spearman rank-order correlation coefficient is a nonparametric assessment of how well the predicted values and actual values can be described using a monotonic function:

$$\rho =\text{1}-\frac{6\sum {\text{d}}_{i}^{2}}{n\left(n^{2}-1\right)};$$

*d*_*i*_: the difference between the ranks of predicted value i and actual value i;

*n*: number of observations.

Like Pearson’s r, Spearman’s ρ varies between -1 to 1. Both extremes indicate a perfect monotonic relationship, while 0 implies no correlation. In Ciclops, Spearman’s ρ is calculated using the *scipy.stats* module in Python.

Users can also implement customized evaluation metrics relevant to their research questions using the results from running Ciclops.

## Limitations

When using Ciclops for cross-platform model building, the model performance will vary based on the training dataset, the test dataset, and the machine learning method being applied. Users may even see model performance vary across different test sets generated in the same study, but under different conditions. These disparities in model performance may be due to the nature of the data collection methods, environmental factors drastically impacting gene expression and/or clinical outcomes, or large biological variability which the model fails to explain. Therefore, users must be critical and careful in their research design before considering the strategy implemented in Ciclops.

Another limitation is that because SHAP analysis works best and fastest with tree-based models, it is not implemented for use with the regression models that can be used in Ciclops (namely, Gaussian Process Regression and Linear Regression). Since transcriptomic data typically contain thousands of genes’ expression levels, it would be too computationally expensive to use SHAP with these regression models. Users are recommended to explore alternative methods of feature importance analysis for these models if they deem it necessary.

Finally, users are also limited in their choice of machine learning algorithms. While the algorithms implemented in Ciclops have been popular in recent years for their top performance, users may wish to implement a different machine learning algorithm of their choice. In this case, users can modify the source code to fit their needs.

## Troubleshooting

### Problem 1

Ciclops failed to install due to uninstalled dependencies (step 1).

### Potential solution

Refer to the key resources table to manually install the packages, paying attention to the minimum version requirement.

### Problem 2

Ciclops fails to run due to clashing dependencies or deprecation warnings (step 1).

### Potential solution

Some of Ciclops’ dependencies have dependencies of their own, which may have differing version requirements.

• If you get a deprecation warning on the command line after calling Ciclops, consult the key resources table to upgrade all of Ciclops’ dependencies to their most recent version, despite the minimum version requirement being lower.
• If you get an error because other packages in your local environment have version requirements of certain dependencies that clash with that of Ciclops, consult the documentation of the packages with clashing dependencies. If you can reinstall a version of the clashing dependency that satisfies both Ciclops and the other package’s version requirements, do so using the command:

pip install [dependency]==[version]

If there is no version overlap between Ciclops and the other package, consider creating a new virtual environment for your project by using *conda* or *virtualenv* for Python 3 and installing all of Ciclops’ dependencies listed in the key resources table in this virtual environment before installing Ciclops.

### Problem 3

**Figure 4:**
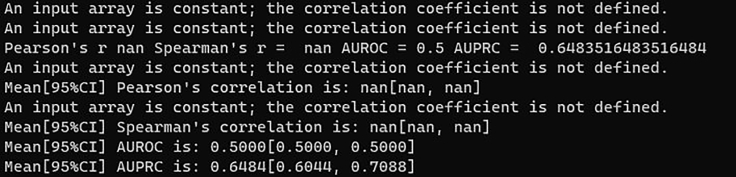
Troubleshooting example Some of the output of Ciclops if the datasets provided to the program do not share any genes.

Ciclops does not produce the expected results because the program cannot find common genes between the training and test set (step 2a, expected outcomes). You may have noticed this because the csv files containing the train/test splits in the ./training directory only have two columns: sample names and labels, missing any gene expression columns; or your output after running Ciclops resembles that of Figure 4.

### Potential solution

• If your datasets have genes in common, but have different naming schemes, ensure the gene names are consistent between the training and test sets before running Ciclops.
• If your datasets do not have genes in common, select different datasets that have genes in common in order to build a predictive model.

### Problem 4

Users wish to use different hyperparameters for the machine learning models implemented in Ciclops.

### Potential solution

We encourage users to tune the hyperparameters of the models they wish to use if they are trying to improve model performance. The hyperparameter settings in Ciclops are listed in Table 3, and can be modified by editing the model.py script in the source code. For more detailed information on parameter options and ranges, please refer to the documentation of the machine learning model you wish to tune.

• As an example, if you are trying to tune the hyperparameters of a LightGBM model, you may experiment with smaller values for ‘num_leaves’ and ‘n_estimators’ if your model is overfitting. Alternatively, you may wish to speed up the training process by increasing the ‘learning_rate’ hyperparameter, or try to achieve higher prediction accuracy by lowering the learning rate.
• Note that the optimal hyperparameters for your model may depend on the characteristics of the datasets you are using; for example, the number of features or samples in your datasets.

### Problem 5

Ciclops does not produce the expected results (expected outcomes). Your results may resemble the output presented in Figure 4, or your results seem too extreme in the statistical sense (for example, confidence intervals spanning [0, 1] for AUPRC, or NaN values for any of the evaluation metrics).

### Potential solution

• Consult the potential solution to problem 3 if you used datasets that did not have any genes in common.
• If your results don’t make sense, examine the datasets to make sure they are not empty.
• Examine your datasets for any outliers, and do some sanity checks on all your features and labels.
• Tune the hyperparameters to obtain a better fit (see problem 4) and try different models that can be used with Ciclops.

## Resource availability

### Lead contact

Further information and requests for resources and reagents should be directed to and will be fulfilled by the lead contact, Yuanfang Guan (gyuanfan@umich.edu).

### Materials availability

This study did not generate any new unique reagents.

## Data Availability

The script to obtain the example datasets from GEO and the code generated in this study are publicly available at https://github.com/GuanLab/ciclops. All original code has been deposited at Zenodo and is publicly available as of the date of publication. The DOI is listed in the key resources table. Any additional information required to reanalyze the data reported in this paper is available from the lead contact upon request.

## References

[bib16] Chen T., Guestrin C. (2016). XGBoost: A Scalable Tree Boosting System. Proceedings of the 22nd ACM SIGKDD International Conference on Knowledge Discovery and Data Mining.San Francisco, California, USA:. Association for Computing Machinery.

[bib17] da Costa-Luis C. (2022). tqdm: A fast, extensible progress bar for Python and CLI. Zenodo.

[bib1] Fan X., Lobenhofer E.K., Chen M., Shi W., Huang J., Luo J., Zhang J., Walker S.J., Chu T.M., Li L. (2010). Consistency of predictive signature genes and classifiers generated using different microarray platforms. Pharmacogenomics J..

[bib2] Fauteux F., Surendra A., McComb S., Pan Y., Hill J.J. (2021). Identification of transcriptional subtypes in lung adenocarcinoma and squamous cell carcinoma through integrative analysis of microarray and RNA sequencing data. Sci. Rep..

[bib3] Guo L., Lobenhofer E.K., Wang C., Shippy R., Harris S.C., Zhang L., Mei N., Chen T., Herman D., Goodsaid F.M. (2006). Rat toxicogenomic study reveals analytical consistency across microarray platforms. Nat. Biotechnol..

[bib4] Harris C.R., Millman K.J., Van Der Walt S.J., Gommers R., Virtanen P., Cournapeau D., Wieser E., Taylor J., Berg S., Smith N.J., Kern R. (2020). Array programming with NumPy. Nature.

[bib5] Hunter J.D. (2007). Matplotlib: a 2D graphics environment. Comput. Sci. Eng..

[bib6] Ke G., Meng Q., Finley T., Wang T., Chen W., Ma W., Ye Q., Liu T.Y., Guyon I., Von Luxburg U., Bengio S., Wallach H., Fergus R., Vishwanathan S., Garnett R. (2017).

[bib7] Lundberg S.M., Lee S.-I., Guyon I., Von Luxburg U., Bengio S., Wallach H., Fergus R., Vishwanathan S., Garnett R. (2017).

[bib8] McKinney W., Van der Walt Stéfan, Millman Jarrod (2010). In proceedings of the 9th Python in Science Conference.

[bib9] Mok S., Ashley E.A., Ferreira P.E., Zhu L., Lin Z., Yeo T., Chotivanich K., Imwong M., Pukrittayakamee S., Dhorda M. (2015). Drug resistance. Population transcriptomics of human malaria parasites reveals the mechanism of artemisinin resistance. Science.

[bib10] Mok S., Stokes B.H., Gnädig N.F., Ross L.S., Yeo T., Amaratunga C., Allman E., Solyakov L., Bottrill A.R., Tripathi J. (2021). Artemisinin-resistant K13 mutations rewire Plasmodium falciparum’s intra-erythrocytic metabolic program to enhance survival. Nat. Commun..

[bib11] Pedregosa F., Varoquaux G., Gramfort A., Michel V., Thirion B., Grisel O., Blondel M., Prettenhofer P., Weiss R., Dubourg V., Vanderplas J. (2011). Scikit-learn: machine learning in Python. J. Machine Learn. Res..

[bib12] Sage Bionetworks. Malaria DREAM Challenge. https://www.synapse.org/#!Synapse:syn16924919/wiki/583955.

[bib14] Virtanen P., Gommers R., Oliphant T.E., Haberland M., Reddy T., Cournapeau D., Burovski E., Peterson P., Weckesser W., Bright J. (2020). SciPy 1.0: fundamental algorithms for scientific computing in Python. Nat. Methods.

[bib15] Zhang H., Guo J., Li H., Guan Y. (2022). Machine learning for artemisinin resistance in malaria treatment across in vivo-in vitro platforms. iScience.

